# *In vitro* characterization of gamma oscillations in the hippocampal formation of the domestic chick

**DOI:** 10.1111/ejn.13773

**Published:** 2018-01-10

**Authors:** Pradeep Dheerendra, Nicholas M. Lynch, Joseph Crutwell, Mark O. Cunningham, Tom V. Smulders

**Affiliations:** ^1^ Institute of Neuroscience Newcastle University Framlington Place Newcastle upon Tyne NE2 4HH UK; ^2^ University of Louisville Louisville KY USA; ^3^ Centre for Behaviour and Evolution Newcastle University Newcastle upon Tyne UK

**Keywords:** avian hippocampus, *Gallus gallus domesticus*, homology, local field potentials, rhythmogenesis

## Abstract

Avian and mammalian brains have evolved independently from each other for about 300 million years. During that time, the hippocampal formation (HF) has diverged in morphology and cytoarchitecture, but seems to have conserved much of its function. It is therefore an open question how seemingly different neural organizations can generate the same function. A prominent feature of the mammalian hippocampus is that it generates different neural oscillations, including the gamma rhythm, which plays an important role in memory processing. In this study, we investigate whether the avian hippocampus also generates gamma oscillations, and whether similar pharmacological mechanisms are involved in this function. We investigated the existence of gamma oscillations in avian HF using *in vitro* electrophysiology in P0–P12 domestic chick (*Gallus gallus domesticus*) HF brain slices. Persistent gamma frequency oscillations were induced by the bath application of the cholinergic agonist carbachol, but not by kainate, a glutamate receptor agonist. Similar to other species, carbachol‐evoked gamma oscillations were sensitive to GABA_A_, AMPA/kainate and muscarinic (M1) receptor antagonism. Therefore, similar to mammalian species, muscarinic receptor‐activated avian HF gamma oscillations may arise via a pyramidal‐interneuron gamma (PING)‐based mechanism. Gamma oscillations are most prominent in the ventromedial area of the hippocampal slices, and gamma power is reduced more laterally and dorsally in the HF. We conclude that similar micro‐circuitry may exist in the avian and mammalian hippocampal formation, and this is likely to relate to the shared function of the two structures.

## Introduction

Avian and mammalian brains have diverged for over 300 million years since their last common ancestor. The telencephalon of these two lineages is very differently organized (Jarvis *et al*., 2005), and there is an ongoing debate about whether the avian dorsoventricular ridge (DVR) is homologous to the mammalian neocortex, or whether they derive from different developmental regions (Striedter *et al*., 2014; Karten, 2015). Functionally, however, they seem to exhibit convergent abilities (Emery, 2004, 2006; Clayton & Emery, 2015). Unlike for cortex, there is a general consensus that the hippocampal formations (HF) of both lineages are homologous to each other, as they both derive from the medial pallium (Striedter, 2016). Although hippocampal function seems conserved in the two lineages, with important roles in memory, spatial navigation and feedback onto the hypothalamic–pituitary–adrenal axis (Kahn & Bingman, 2009; Mayer *et al*., 2013; Herold *et al*., 2015; Smulders, 2017), the anatomical organization of the two structures differs dramatically. Whereas the mammalian hippocampal formation has clearly delineated subdivisions such as the entorhinal cortex, subiculum, Ammon's horn regions and dentate gyrus (Andersen, 2007), the avian HF has no such clear cell fields (Gupta *et al*., 2012; Herold *et al*., 2014; Striedter, 2016). Different subdivisional homologies have been proposed between avian and mammalian hippocampal formations, but the debate is still ongoing (Striedter, 2016). The existence of such functionally similar, yet anatomically dissimilar structures between birds and mammals leads us to the question: How do such divergent structures perform such similar functions?

Much has been made about the macroscopic organization of the mammalian HF (and indeed the cortex) in discrete cell layers with regard to its function (Andersen, 2007), but the fact that the avian HF can carry out the same functions without the clearly delineated cell layers suggests that this is not the case. However, it is still possible that a similar connectional organization underlies the divergent macroscopic organizations to deliver a similar function (Striedter, 2016). Again, connections between avian hippocampal subdivisions have been studied and different hypotheses put forward about the similarities and differences between the avian and mammalian HF (Szekely, 1999; Hough *et al*., 2002; Kahn *et al*., 2003; Atoji & Wild, 2004, 2006). However, to date, nobody has yet studied local circuit functionality in the avian HF and compared it to that in the mammalian hippocampus. The study we present here aims to make a start at better understanding local circuit properties in the avian HF and relate these to what we know about local circuit properties in the mammalian system.

One prominent feature of the mammalian hippocampus is its ability to generate discrete forms of neuronal oscillations. In particular, gamma rhythms (30–80 Hz) are a physiological feature of the mammalian hippocampus (Traub *et al*., 1996), which play an important role in memory processing. They are posited to provide a substrate for organization and binding of different perceptions (Singer, 1996). *In vitro* studies using pharmacologically driven models (e.g. application of carbachol or kainate) of gamma oscillations have revealed the circuitry responsible for this activity in mammals. While pyramidal neurons fire sporadically during gamma oscillations (Fisahn *et al*., 1998; Gloveli *et al*., 2005), in contrast, fast‐spiking parvalbumin‐expressing interneurons fire on each gamma cycle and are strongly phase locked to the oscillation (Gloveli *et al*., 1997; Hajos *et al*., 2004). Further studies have demonstrated that these perisomatic targeting interneurons are critical for the generation of gamma oscillations in the rodent CA3 region (Oren *et al*., 2006, 2010; Mann & Mody, 2010). As pyramidal–interneuron network gamma oscillations require phasic excitatory synaptic input onto interneurons, it is unsurprising that the selective reduction in GluA1‐ or GluA4‐containing AMPA receptors on hippocampal interneurons (Fuchs *et al*., 2001, 2007) has profound implications for network gamma oscillations. It is this negative feedback loop (Wang & Buzsáki, 1996) between pyramidal neurons and parvalbumin‐expressing fast‐spiking interneurons (Freund & Buzsáki, 1996) that generates the gamma oscillations in mammals. Gamma rhythm generation in the mammalian HF is most prominent in the CA3 region (Csicsvari *et al*., 2003) and is communicated to other regions of the HF and beyond from there.

The existence of gamma oscillations has not yet been explored in the avian HF. They have, however, been described in the avian optic tectum (OT) in the midbrain (Sridharan & Knudsen, 2015). In barn owls (*Tyto alba*), a brief visual stimulation that engages the bird's attention triggers a brief gamma oscillation in the OT (Sridharan *et al*., 2011). This can be mimicked *in vitro* in OT slices taken from poultry chicks (*Gallus gallus domesticus*) by providing a brief electrical stimulation to the region that receives retinal inputs (Goddard *et al*., 2012). The local OT circuit generating these oscillations depends on GABA_A_ receptors (both to generate the rhythm and to set the peak frequency of this activity), NMDA glutamate receptors (to prolong the oscillation following termination of the stimulus) and on acetylcholine receptors. The GABAergic neurons involved in the gamma‐generating circuitry seem to be parvalbumin^+^ inhibitory interneurons (Goddard *et al*., 2012), as is also the case for mammalian CA3 gamma oscillations (Cardin *et al*., 2009; Sohal *et al*., 2009).

It is clear that avian brains can generate gamma oscillations, just like mammalian brains can. In this study, the questions we aim to address through *in vitro* electrophysiology are whether (i) the avian HF also contains local circuits that generate and sustain gamma frequency oscillations, (ii) these HF micro‐circuits exhibit pharmacological similarities to the gamma‐generating circuits in the mammalian hippocampus, and (iii) there is a subregional differentiation in the generation of gamma rhythms in the avian HF.

## Materials and methods

### Animals

For the pharmacological characterization, Welsummer breed chicken eggs (*Gallus gallus domesticus*) were obtained from a local breeder and incubated in the Comparative Biology Centre, Newcastle University. A total of 17 birds were used for this study. Upon hatching, we housed them as a group in a heated cage, provided with food and water *ad libitum*, and subjected to a regular 12‐h light/dark cycle (0800‐2000 hours).

For the spatial mapping, we obtained 1‐day‐old broiler chicks (Ross 308) from a commercial hatchery in Thirsk, UK, which provided a more reliable supply of birds. A total of 18 birds were used in this study. They were transported to the laboratory by car, where they were housed in floor pens measuring 120 × 70 cm. The floor of the pens was covered with wood chips, and each pen contained a food hopper, a water hopper and a plastic shelter. Chicks were subject to a 14L : 10D cycle using uncovered fluorescent lights, and the temperature of the laboratory was maintained at 26–30 °C using room heaters. Water was provided *ad lib*., as were chick starter crumbs. Chicks also received mealworms *Tenebrio molitor* once a day in their home cages. All subjects were marked with non‐toxic ‘child‐friendly’ coloured marker pens, and their weights were monitored daily for welfare purposes.

To obtain brains for slice preparation, we anaesthetized chicks aged P0–P12 days (of either sex) by the brief inhalation of isoflurane (0.05%) and euthanized them by cervical dislocation. We carried out all procedures in accordance with UK Home Office guidelines set out in the Animals (Scientific Procedures) Act 1986. The protocol was approved by Newcastle University's Animal Welfare and Ethical Review Board (Project ID No 380).

### Slice preparation

Following decapitation, the brain was immediately removed and placed in ice‐cold (~0 °C) sucrose‐based artificial cerebrospinal fluid (ACSF) solution composed of the following (in mm): sucrose, 25; D‐glucose, 10; NaHCO_3_, 24; KCl, 3; MgCl_2_, 5; CaCl_2_, 2; NaH_2_PO_4_, 1.25; MgSO_4_, 2, continuously bubbled with carbogen (95% O_2_, 5% CO_2_). The forebrain was then mounted on a steel plate, and 400‐μm thin coronal sections were cut in the cold, sucrose‐based ACSF solution using a Leica VT1000 microtome (Leica Microsystems, Germany). The hippocampus was then dissected free from the surrounding brain regions and transferred to a holding chamber containing ACSF at room temperature. For recordings, slices were then transferred to an interface style chamber and maintained at the interface containing standard ACSF, composed of the following (in mm): NaCl, 126; KCl, 3; NaH_2_PO_4_, 1.25; NaHCO_3_, 24; MgSO_4_, 1; CaCl_2_, 1.2; glucose, 10. The circulating ACSF solution was continuously bubbled with humidified carbogen (95% O_2_, 5% CO_2_) to maintain a pH of 7.4 and warmed to 34 ± 1 °C. Slices were allowed to equilibrate for at least 30 min prior to any recording.

### Recording and data acquisition

Glass microelectrodes for extracellular local field potentials (LFP) recordings were pulled from thin‐walled borosilicate tubing (1.2 mm O.D. × 0.94 I.D., Harvard Apparatus Ltd., Kent, UK) to a resistance of 2–5 MΩ using a PP‐83 Narishige puller (Narishige Scientific Instrument Lab, Tokyo, Japan) and filled with standard ACSF. Up to three microelectrodes were then placed in pipette holders (World Precision Instruments, FL, USA) attached to pre‐amplifier headstages (HS‐2A, Axon Instruments Inc., Union City, CA, USA) mounted to manual micromanipulators (Narishige Scientific Instrument Lab, Tokyo, Japan). Data were recorded in a current‐clamp mode using an AI2130 differential amplifier (Axon Instruments Inc.) and band‐pass filtered at 10–100 Hz using an external 8‐pole Bessel filter (Applegarth Electronics, Oxford, UK). Data were then digitized at 10 kHz by a ITC‐18 A/D convertor (Heka, Harvard Bioscience). Mains noise (50 Hz) was removed using Humbugs (Quest Scientific Instruments Inc., North Vancouver, Canada). Data were recorded using axograph x software (Axograph Scientific, Sydney, Australia) on an Apple Mac computer (Apple Computers Inc., CA, USA).

Following the application of carbachol to the circulating ACSF to induce gamma oscillations, the network activity was allowed to stabilize before continuing with any further manipulations. Stabilization is categorized as there being no more than a 10% change in the value of both amplitude and power in three successive 60s recordings taken at 10‐min intervals. For the pharmacological manipulations, the electrode was placed in the centre of the hippocampal slice. For the subregional mapping of gamma power, the electrode was moved around in the slice to seven different locations (see Results). The order in which the different locations were recorded from was different for each slice. The electrode was allowed to rest in each regional position for 60 s before any data were recorded.

### Electrophysiology data analysis

Power spectra of the recorded LFP were calculated from 60‐s epochs of recorded field activity using a fast Fourier transform (FFT) in the axograph x software package. We quantified it by extracting frequency and amplitude corresponding to peak in the gamma band of the power spectrum, while area power was determined as the area within 20 and 80 Hz. This gave us peak power (μV^2^/Hz) at an oscillation frequency (Hz) as well as the area power (total power of the oscillation (μV^2^/Hz·kHz) in the gamma frequency band.

### Pharmacological manipulations

All drugs were bath applied at known concentrations previously used in rodent brain slice experiments: Kainic acid [(2S,3S,4S)‐3‐carboxymethyl‐4‐(prop‐1‐en‐2‐yl)pyrrolidine‐2‐carboxylic acid], 50–800 nm; Carbachol [2‐[(Aminocarbonyl)oxy]‐N,N,N‐trimethylethanaminium chloride], 10 μm (unless otherwise indicated); Physostigmine [(3aR,8aS)‐1,3a,8‐Trimethyl‐1H,2H,3H,3aH,8H,8aH‐pyrrolo[2,3‐b]indol‐5‐yl N‐methylcarbamate], 10 μm; NBQX [2,3‐dioxo‐6‐nitro‐1,2,3,4‐tetrehydrobenzo[*f*]quinoxaline‐7‐sulfonamide], 20 μm; Gabazine [4‐[6‐imino‐3‐(4‐methoxyphenyl)pyridazin‐1‐yl] butanoic acid hydrobromide], 1 μm; Atropine [(RS)‐(8‐Methyl‐8‐azabicyclo[3.2.1]oct‐3‐yl) 3‐hydroxy‐2‐phenylpropanoate], 1 μm; Scopolamine [(–)‐(S)‐3‐Hydroxy‐2‐phenylpropionic acid (1R,2R,4S,7S,9S)‐9‐methyl‐3‐oxa‐9‐azatricyclo‐[3.3.1.02,4]non‐7‐yl ester], 10 μm; Pirenzepine [11‐[(4‐methylpiperazin‐1‐yl)acetyl]‐5,11‐dihydro‐6H‐pyrido[2,3‐b][1,4]benzodiazepin‐6‐one], 1 μm; D‐AP5 [2‐amino‐5‐phosphonopentanoic acid], 50 μm. All were obtained from Tocris Cookson (Bristol, UK). Salts for ACSF and sucrose‐ACSF were from BDH‐Merck.

### Immunohistochemistry

Brains were removed using the same method as explained above, but then placed into 4% Paraformaldehyde solution immediately after dissection before being transferred to 30% sucrose solution after 2 days for cryoprotection. Brains were then submerged in optimal cutting temperature (OCT) compound and cut to 40 μm on a MICROM HM560 cryostat before being stored at 0–4 °C in PBS. Sections were exposed to primary mouse anti‐parvalbumin antibody (Sigma p3088) at 1 : 2000 concentration in PBS‐1% Triton X‐100 and 3% Horse serum and left to incubate overnight at 4 °C. The secondary horse anti‐mouse antibody was applied at room temperature for 2 h at 1 : 200 concentration in PBS. Sections were incubated in horseradish peroxidase (Ready‐To‐Use Streptavidin Horseradish Peroxidase, Vector Labs, UK) for 2 h at room temperature. The chromogenic reaction was then developed using DAB (SigmaFast^®^ DAB tablets, Sigma‐Aldrich, UK) for 5 min. Sections were mounted onto gelatine‐subbed glass slides (Fisher Scientific, UK). After air‐drying overnight, slides were washed in distilled water and sections were then dehydrated by incubation in sequential ethanol concentration gradients; starting from 70, 95 and 100%; each for 5 min each, with a further 5 min in another 100% ethanol gradient. Sections were then bathed in Histoclear (National Diagnostics, USA) for 5 min twice before being coverslipped using Eukitt^®^ (Sigma‐Aldrich, UK).

### Statistical analysis

Statistical analyses were performed in IBM spss v.23. Gamma oscillations are analysed as the Area Under the Curve power in the gamma range (20–80 Hz). Because of large variation in absolute values from experiment to experiment, the data were expressed as a percentage of the value before the treatment started and then log_10_‐transformed. For the regional mapping, data were expressed as a percentage of the ventromedial region power and log_10_‐transformed. Data are analysed with paired *t* tests when comparing two treatments, or repeated‐measures anova when more than two treatments are compared. For the regional mapping, we used the Generalized Estimating Equations function in spss, because it can run with missing data, which the repeated‐measures anova function cannot. The output from this analysis is reported as Wald's χ^2^. Descriptive statistics are expressed as Mean ± Standard Error of the Mean.

## Results

### Gamma induction in avian hippocampal slices

Low concentrations of the kainate receptor agonist kainic acid are known to induce robust oscillations in mammalian hippocampus and associated neocortical structures. Interestingly, 100 nm of kainic acid was unable to elicit gamma activity in the avian HF (on average 1.46 (±1.42) times the area power of baseline, *t*_7_ = 1.09, *P = *0.31; without any change in peak frequency, *t*_7_ = 0.40, *P = *0.70; *n* = 8 slices from three birds). In three of these slices (from one bird), the concentration of kainic acid was slowly increased to 800 nm, but no significant change in gamma power ensued (on average 3.11 (±1.57) times baseline area power, *t*_2_ = 1.54, *P = *0.26; no change in peak frequency, *t*_2_ = 0.16, *P = *0.89).

**Figure 1:**
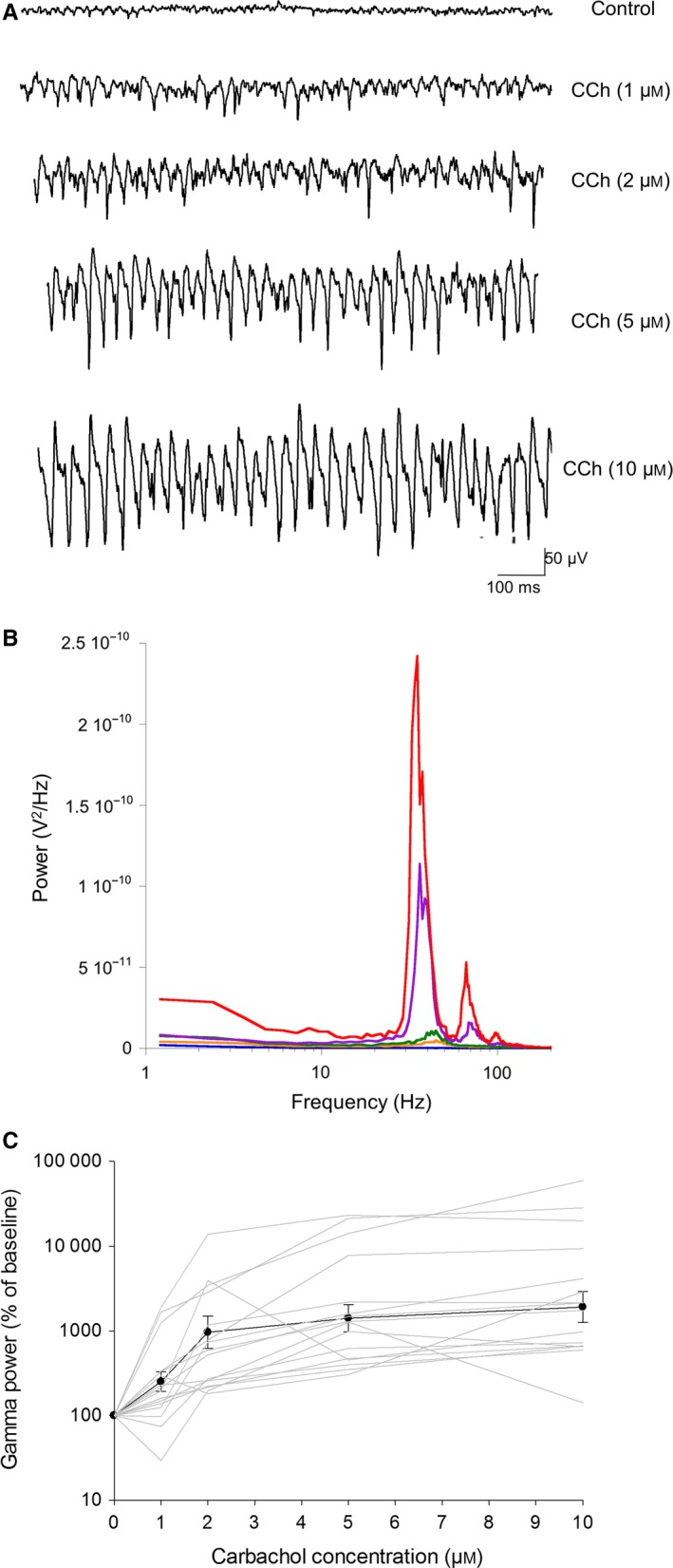
(A) Example induction of gamma oscillations using different concentrations of carbachol in the same slice. (B) Spectrograms of gamma oscillations. Different colours represent different concentrations of carbachol: black: 0 μm; orange: 1 μm; green: 2 μm; purple: 5 μm; red: 10 μm. (C) Carbachol significantly increases the power (area under the curve) in the gamma band in a dose‐dependent measure. There is a significant induction of gamma oscillations with 1 μm of carbachol and a significant increase in power with 2 μm. The increase from 2 to 10 μm is not statistically significant due to high variability among slices, as indicated by the grey lines, which each represent a different slice.

As we had no immediate success with kainic acid, we examined the impact of carbachol, a broad‐spectrum cholinergic agonist, which also induces robust gamma oscillations in mammalian hippocampal slices (Fisahn *et al*., 1998). Unlike kainic acid, carbachol induced robust gamma oscillations in the avian hippocampal formation, in a dose‐dependent manner (Fig. 1; *F*_4,12_ = 18.14, *P < *0.001, *n* = 16 slices from six birds). On average, 10 μm carbachol led to an area power that was 22.80 (±1.51) times the baseline area power, with a mean peak frequency of 36 ± 1.4 Hz. Once initiated, the network activity was stable for few hours (2–3 h tested).

**Figure 2:**
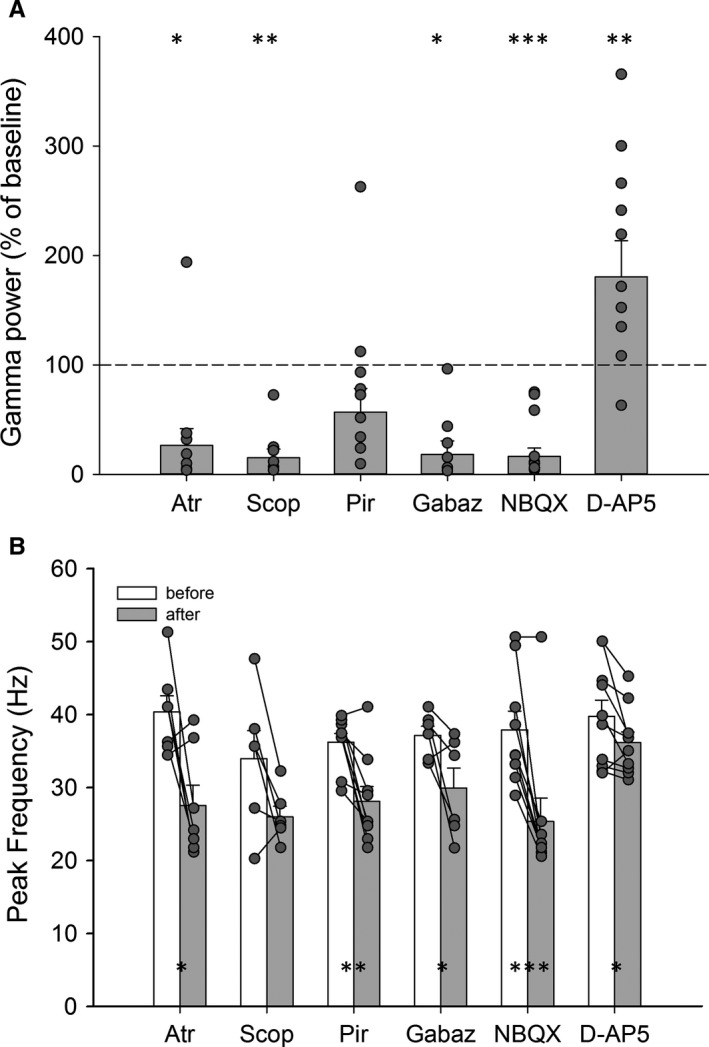
(A) Change in area under the curve gamma power after treatment with the different pharmacological agents, expressed as a percentage of the gamma power before adding the drug. Individual points represent separate slices, while the bars represent means + SEM. (B) Change in peak frequency after treatment with the different pharmacological agents. Atr = 1 μm Atropine; Scop = 10 μm Scopolamine; Pir = 1 μm Pirenzepine; Gabaz = 1 μm Gabazine; NBQX = 20 μm NBQX; D‐AP5 = 50 μm D‐AP5. *P < 0.05; **P < 0.01; ***P < 0.001. Connected points represent the change within one slice. Bars represent means + SEM.

To investigate whether muscarinic cholinergic receptors play a role in the rhythmogenesis of gamma oscillations, we tested the effect of atropine, a generic muscarinic antagonist. After inducing gamma oscillations using 10 μm of carbachol, bath application of 1 μm of atropine eliminated the oscillations (Fig. 2; 18.5% of carbachol‐induced area power, *t*_6_ = 3.27, *P = *0.017; reduction in peak frequency from 40 ± 2.2 to 28 ± 2.8 Hz, *t*_6_ = 3.03, *P = *0.023; in seven slices from three animals in which the area power increased again after atropine washout). Further, we aimed to investigate the role of M1 subtype of the muscarinic receptors. Bath application of 10 μm of scopolamine, an M1‐specific antagonist, inhibited the carbachol‐induced gamma oscillations (15% of carbachol‐induced area power, *t*_5_ = 4.44, *P = *0.007, *n* = 6 slices from animals in which area power increased again upon washout). There was an almost significant drop in peak frequency from 34 ± 3.8 to 26 ± 1.5 Hz (*t*_5_ = 2.51, *P = *0.054; Fig. 2). This was confirmed with bath application of 1 μm of pirenzepine, another M1‐subtype‐specific muscarinic antagonist. Area under the curve did not significantly decrease (on average 57% of carbachol‐induced gamma power, *t*_8_ = 1.76, *P = *0.12, *n* = 9 slices from two animals in which area power increased after washout of pirenzepine), but the peak frequency did drop below the gamma range (from 36 ± 1.2 to 28 ± 2.0 Hz, *t*_8_ = 3.94, *P = *0.004; Fig. 2).

### Involvement of GABAergic inhibition and Glutamatergic excitation?

Gamma activity in the mammalian hippocampus is known to be critically dependent on the rhythmic output of populations of GABAergic interneurons (Whittington *et al*., 1995). To test whether this hypothesis held in the avian HF, we first induced gamma activity with 10 μm of carbachol. Addition of 1 μm of Gabazine, a specific GABA_A_ receptor antagonist, resulted in almost complete abolition of the gamma activity (18% of carbachol‐induced area power, *t*_5_ = 3.30, *P = *0.023; *n* = 6 slices from four animals in which area power increased after washout), reducing the peak frequency from 37 ± 1.3 to 30 ± 2.8 Hz (*t*_5_ = 2.58, *P = *0.05; Fig. 2).

Fast phasic glutamatergic transmission has been demonstrated to influence persistent gamma activity in the mammalian hippocampus (Whittington *et al*., 2000; Bartos *et al*., 2007). Thus to investigate the role of AMPA receptors in the avian HF, we tested the effect of NBQX, an AMPA/kainate receptor antagonist. We again induced gamma oscillations by bath application of 10 μm of carbachol. Application of 20 μm of NBQX resulted in complete abolition of the gamma activity (16% of carbachol‐induced area power, *t*_8_ = 4.74, *P = *0.001, *n* = 9 slices from four animals; Fig. 2). The peak frequency is also significantly reduced from 38 ± 2.6 to 25 ± 3.2 Hz (*t*_8_ = 4.93, *P = *0.001). Finally, to investigate the role of NMDA receptors in the gamma rhythmogenesis, we tested the effect of D‐AP5, an NMDA receptor antagonist, on the gamma activity induced by 10 μm of carbachol. After inducing gamma oscillations by a bath application of carbachol, we bath applied 50 μm of D‐AP5. Interestingly, whereas D‐AP5 slightly reduced the peak frequency from 40 ± 2.2 to 36 ± 1.4 Hz (*t*_9_ = 2.53, *P = *0.032), the area power in the gamma range significantly increased (1.8 times the carbachol‐induced area power, *t*_9_ = 3.54, *P = *0.006, 10 slices from five animals; Fig. 2).

### Mapping of gamma power in the different subdivisions

**Figure 3:**
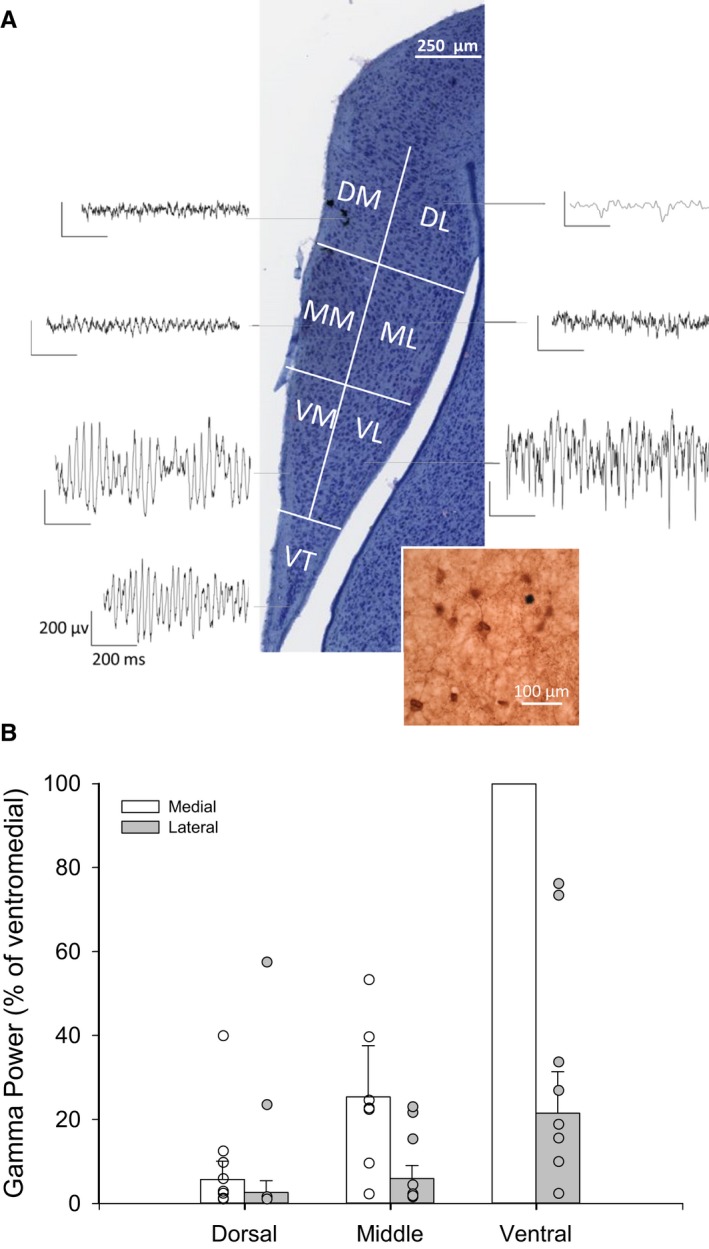
(A) Example of distribution of gamma power around an anterior coronal hippocampal slice from the chicken brain (image from Brainmaps.org). The inset shows parvalbumin‐positive cells in the chick hippocampal formation. VT, ventral tip; VM, ventromedial; VL, ventrolateral; MM, middle medial; ML, middle lateral; DM, dorsomedial; DL, dorsolateral. (B) Area under the curve gamma power, normalized to the power in the ventromedial region. Power is lower as the electrode is moved both from medial to lateral and from ventral to dorsal. Ventral tip was left out of this analysis (see text). Individual points represent separate slices. Although the analysis was performed as a repeated‐measures analysis, the points derived from the same slices have not been connected with each other to keep the graph simple.

We recorded the gamma power in six different areas in the slice and analysed these with a Generalized Estimating Equations approach. Gamma power was highest in the ventromedial region and became progressively weaker as the electrode was moved dorsally ($\chi_{2}^{2}$ = 35.32, *P < *0.001) and laterally ($\chi_{1}^{2}$ = 12.41, *P < *0.001; nine slices from six animals; Fig. 3). There was no interaction between the two spatial axes ($\chi_{2}^{2}$ = 2.71, *P = *0.26). For the four slices where we were able to record in the ventral‐most tip of the slice, the gamma power was also lower than in the ventromedial region (*t*_3_ = 3.52, *P = *0.039).

### Parvalbumin staining

The anti‐parvalbumin antibody clearly stained a population of PV^+^ cells throughout the hippocampal formation (Fig. 3A inset).

## Discussion

Our main finding is that, like the mammalian hippocampus, the avian hippocampal formation is capable of endogenously generating gamma oscillations when stimulated with a cholinergic agonist. In terms of peak frequency (c. 40 Hz), this cholinergic rhythm in avian slices is similar to the peak frequency of gamma observed in a number of pharmacologically induced (e.g. by kainate, acetylcholine receptor agonists, or metabotropic glutamate receptor agonists) gamma oscillations in the mammalian hippocampus (Fisahn *et al*., 1998, 2004; Palhalmi *et al*., 2004; Fisahn, 2005). Both fast inhibition mediated by GABA_A_ receptors and fast excitation via predominantly AMPA receptors are a requirement for generating and maintaining this oscillation. This suggests that similar functional micro‐circuitry, with similar pharmacological properties, may be present in the avian HF as in its mammalian counterpart, despite a clearly different cytoarchitecture of the structures. Further studies looking at single cells and their connections will allow us to fully characterize this circuitry.

### Similarities to mammalian gamma oscillations

Gamma oscillations were robustly induced using a direct cholinergic agonist. This suggests that there must be a natural cholinergic input into the avian HF that may contribute to the generation of gamma oscillations *in vivo*. The cholinergic antagonists we used suggest a necessary involvement of M1 muscarinic receptors in this process. This finding mimics observations made in the mouse hippocampus in which gamma oscillations were absent in M1 receptor‐deficient mice (Fisahn *et al*., 2002). It is different, however, from the brief gamma oscillations triggered in the chick midbrain, which depend on nicotinic, but not muscarinic acetylcholine receptors (Bryant *et al*., 2015). Interestingly, in adult pigeons, the density of M1 receptors is very low throughout the hippocampus (Kohler *et al*., 1995; Herold *et al*., 2014). Nevertheless, blocking of M1 receptors by intramuscular injection of scopolamine interferes with short‐distance homing in pigeons, a task known to rely on hippocampal‐dependent spatial memory (Kohler *et al*., 1996). It is therefore possible that mAchRs are only expressed by a small, but crucial population of neurons [e.g. on excitatory neurons (Fisahn *et al*., 2002)] and that activation of these receptors would be sufficient to indirectly activate interneurons to produce gamma activity.

The fact that a cholinergic agonist can induce gamma oscillations in the avian HF suggests that there must be an innate cholinergic innervation of the HF. In mammals, there is a strong cholinergic innervation from the septum into the hippocampus (Hasselmo & Bower, 1993). In birds as well, the HF and the septal nuclei are reciprocally connected (Atoji & Wild, 2004; Montagnese *et al*., 2004), but the density of the cholinergic innervation of the HF is lower and originates mainly in the nucleus of the diagonal band (Krayniak & Siegel, 1978; Krebs *et al*., 1991; Montagnese *et al*., 2004).

The circuit that generates the gamma rhythms includes both excitatory and inhibitory synapses, as indicated by the interruption of the oscillations by both a GABA_A_ receptor antagonist and an AMPA receptor antagonist. This suggests that a similar circuit to the proposed pyramidal‐interneuron gamma (PING) oscillator may also exist in the avian hippocampal formation. The presence of parvalbumin^+^ cells in the avian HF is consistent with this, although further work needs to be done to ascertain whether they are also fast spiking, as is the case in the mammalian hippocampus (Freund & Buzsáki, 1996; Gloveli *et al*., 1997; Hajos *et al*., 2004). Indeed, circuits of local inhibitory interneurons and pyramidal projection neurons have been described in both the chick and pigeon hippocampus using Golgi staining methods (Tombol *et al*., 2000a,b). A similar circuit also produces gamma oscillations in the chick midbrain (Goddard *et al*., 2012). However, in those oscillations, which were triggered by a brief electrical stimulus to the slice, D‐AP5 reduced the duration of the gamma oscillations, implying that they are dependent on NMDA receptors for their maintenance (Goddard *et al*., 2012; Sridharan & Knudsen, 2015). The carbachol‐induced gamma oscillations in the hippocampus were not reduced by D‐AP5, however. To the contrary, the power in the gamma band slightly increased, even though the peak frequency was slightly decreased (but remained in the gamma band). This is consistent with carbachol‐induced gamma oscillations in rodent hippocampus, which are also not dependent on NMDA receptors (Fisahn *et al*., 1998). We do not know what may have caused the slight (but significant) increase in gamma power, but similar effects have been seen in hippocampal and cortical gamma oscillations *in vivo* (Pinault, 2008; Lazarewicz *et al*., 2010).

The strongest gamma power by far was recorded in the most ventromedial region of the rostral HF, which is often referred to as Vm (Herold *et al*., 2014). This region has been compared by some to CA3, and by others to the DG of mammals, if any such equivalences can be made at all (Striedter, 2016). What is sure is that in terms of the distribution of different synaptic neurotransmitter receptors (glutamatergic, GABAergic, cholinergic, noradrenergic, serotonergic and dopaminergic), this region is most similar to CA3 (Herold *et al*., 2014). The distribution of receptor binding sites is probably a poor feature to use to derive evolutionary homologies between brain areas, as it is very sensitive to selection pressures that might change the function of a cell population. For that same reason, however, it is a good proxy for functional equivalences, and it is therefore not surprising that the area in the avian HF with the highest pharmacological similarity to CA3 is also the region that generates endogenous gamma rhythms, as does CA3 in mammals.

Of course, in mammals, other regions within the limbic system are also known to be capable of independently generating gamma activity. Subfields of the hippocampus such as the dentate gyrus (Poschel *et al*., 2002; Towers *et al*., 2002), CA1 (Traub *et al*., 2003; Middleton *et al*., 2008; Pietersen *et al*., 2014; Craig & McBain, 2015) and the subiculum (Colling *et al*., 1998; Eller *et al*., 2015) have been demonstrated to be capable of acting as independent gamma generators. In the rodent parahippocampal region, the medial entorhinal cortex has also been shown to be a potent source of gamma frequency oscillations (Cunningham *et al*., 2003; Middleton *et al*., 2008; Colgin *et al*., 2009) capable of entraining network oscillations in CA1. Further work therefore remains to be done to pinpoint the areas in the avian HF that generate gamma oscillations, and those that follow them.

Given the ubiquity of gamma oscillation generators throughout the mammalian brain (Headley & Paré, 2017), and given the existence of gamma oscillations in the avian midbrain (Sridharan & Knudsen, 2015), it is very likely that gamma oscillations can be generated in other parts of the avian forebrain as well. This remains to be explored. Interestingly, however, within the hippocampal formation, data from the spatial studies (Fig. 3) would indicate a localized network in the ventral‐medial avian HF capable of supporting gamma oscillations. Data are now emerging from rodent studies, which have demonstrated coupling of place cell firing to gamma oscillations (Lasztoczi & Klausberger, 2016). Previous studies in freely moving homing pigeons to determine the presence of spatially responsive neurons have only reported unstable firing fields (Siegel *et al*., 2002, 2005, 2006; Bingman *et al*., 2003; Hough & Bingman, 2004, 2008; Kahn *et al*., 2008). Interestingly, the recordings in these studies were conducted in more lateral areas of the HF. Therefore, it may be the case that medial structures of the avian HF, which we have shown to be potent gamma‐generating regions, are the site of location for avian place cells. This hypothesis, of course, remains to be tested.

### Differences from mammalian gamma oscillations

This present study did reveal differences as compared to the mammalian hippocampal slice preparation, however. Unlike in mammalian brain slices (Hajos *et al*., 2000; Fisahn *et al*., 2004; Cunningham *et al*., 2006), kainate was not able to induce gamma oscillations in the avian hippocampus. If this is indeed a feature of the avian HF in general, this may indicate a difference in the conditions that may trigger gamma oscillations *in vivo*. In mammals, kainate‐induced gamma oscillations arise from the influence of kainate receptors (KARs) on interneuronal and excitatory cell function. Activation of KARs on both somatodendritic and axonal compartments of rodent hippocampal interneurons will lead to depolarization (Semyanov & Kullmann, 2001). The increase in interneuron axonal excitability is believed to regulate inhibitory transmitter release (Ali *et al*., 2001). KAR activation also modulates glutamate neurotransmission, particularly at mossy fibre terminals (the main input from the dentate gyrus to CA3) in the rodent hippocampus. During high‐frequency transmission, KARs permit frequency‐dependent facilitation of the mossy fibre input to CA3 pyramidal neurons. As the activity of mossy fibres strongly influences CA3 network activity, this facilitation may be important for regulating the profound feed‐forward inhibition present in CA3 circuitry. Concurrently, activation of KARs located post‐synaptically on pyramidal neurons can promote the excitatory state of these cells via slow excitatory post‐synaptic currents and/or the inhibition of the after‐hyperpolarizing potassium current (I_AHP_), among other inputs (Melyan *et al*., 2002). Birds are thought not to have a functional equivalent of the DG (Bingman & Muzio, 2017), and therefore of the mossy fibres, although they may well have a developmentally homologous region (Abellan *et al*., 2014; Striedter, 2016). It is therefore possible that the kainate synapse that facilitates gamma rhythmogenesis in mammals has never evolved in birds.

Alternatively, it is possible that the inability of kainate to trigger gamma oscillations in chick hippocampus is a developmental, rather than an evolutionary, difference. Although it has not been studied specifically in the hippocampus, the expression of kainate receptors in both the optic tectum (Gomez‐Barriocanal *et al*., 1982) and the cerebellum (Miralles *et al*., 1990; Voukelatou *et al*., 1996) is still increasing dramatically over the first 10 days post‐hatching in chicks. If this is the case in the hippocampus as well, it is possible that kainate expression was not mature enough in the few slices in which we attempted to induce gamma oscillations using kainate. A similar phenomenon is found in rats, in which kainate is unable to induce gamma oscillations in hippocampal slices before post‐natal day 5 (Tsintsadze *et al*., 2015).

### Implications for brain evolution

The discovery of robust gamma oscillations in the avian HF *in vitro* confirms that the avian and mammalian hippocampal formations may well share functional micro‐circuitry. It also reinforces the idea that certain rhythmic patterns are important for proper functioning of brains, and the frequencies of these rhythms are found across a range of species (Buzsaki *et al*., 2013). It is difficult at this point in time to tell whether the similarity in pharmacology implies similar micro‐circuits, and whether these micro‐circuits are homologous or the result of convergent evolution, but they strongly suggest that in order for brain areas to carry out similar behavioural functions, they also need similar physiological functions (and their underlying circuitry). Our first descriptions of gamma oscillations in the avian HF open up a great number of new questions. On the one hand, they beg the question of whether avian hippocampal gamma is triggered under the same conditions *in vivo* as it is in mammals and, therefore, whether gamma oscillations play a similar role of synchronizing populations of neurons with each other for efficient communication (Colgin, 2016). On the other hand, we have only started to scratch the surface of the actual structure of the local circuits in the HF that generate these (and other) hippocampal rhythms. Further work combining pharmacology, patch‐clamp recordings and detailed cellular anatomy will be required to be able to compare the mammalian and avian local circuits in detail. In the mammalian brain, gamma rhythms are generated by different circuitry in different brain areas. Comparing mammalian and avian hippocampal circuits will give us deeper insights into the evolution of these circuits and how they relate to the specific functions of the brain areas in which they occur.

## Conclusion

We have shown that the avian hippocampal formation can generate gamma oscillations *in vitro* and that these share a number of features with similar oscillations in the mammalian hippocampus. The very existence of local circuits for generating gamma oscillations in the avian HF points to similarity in physiological processes and features that are required for similar information processing functions that exist in the avian and mammalian structures.

## Conflict of interest

The authors declare that there are no conflict of interests.

## Author contributions

MOC and TVS designed the study and trained the others in data collection. PD, NML and JC collected the data. PD, JC and TVS analysed the data. PD and TVS wrote the first draft of the manuscript, and the other authors commented on subsequent versions. All authors approved the submission of the final manuscript.

## Data accessibility

Data used in the statistical analyses are uploaded with the manuscript. Raw data files are archived on the laboratory servers and are available upon request.
