# Supplementary material for: In vitro characterization of gamma oscillations in the hippocampal formation of the domestic chick
Source: Eur J Neurosci. 2018 Jan 10;48(8):2807–15. doi: 10.1111/ejn.13773 (PMC6220815; doi:10.1111/ejn.13773)
Supplement: Supplementary file 1 [file EJN-48-2807-s001.pdf]

EJN European Journal of Neuroscience FENS Federation of European Neuroscience Societies

In-vitro characterization of gamma oscillations in the hippocampal formation of the domestic chick

Pradeep Dheerendra, Nicholas M. Lynch, Joseph Crutwell, Mark O. Cunningham & Tom V. Smulders

Review timeline:

Submission date:	09 August 2017
Editorial Decision:	11 September 2017
Revision received:	28 October 2017
Accepted:	02 November 2017

Editor: John Foxe

1st Editorial Decision 11 September 2017

Dear Dr. Smulders,

Your manuscript was reviewed by two external reviewers as well as by the editors. The reviews collectively indicate that your experiments generated new and important information and all concerned found the paper to be a very compelling piece of work, well written and clearly articulated. However, there are several (mostly minor) issues that need to be resolved before we can proceed further with your manuscript for publication in EJN. The reviewers have provided clear points to which you should respond in your revision. In addition, please also attend to the following issues which have been identified by the editorial office:

1) Be sure to provide the full name of the ethics committee that approved the work
2) Please specify the % isoflurane used to anesthetize chicks prior to decapitation
3) Please be very explicit about the number of animals used in the experiment
4) A scale bar is needed for fig 3a
5) Replace your barcharts with scatterplots or some other more informative hybrid depictions - see http://onlinelibrary.wiley.com/doi/10.1111/ejn.13400/epdf

If you are able to respond fully to the points raised, we would be pleased to receive a revision of your paper within 30 days.

Thank you for submitting your work to EJN.

Kind regards,

John Foxe & Paul Bolam
co-Editors in Chief, EJN

Reviews:

Reviewer: 1 (Verner Bingman, Bowling Green State University, USA)

Comments to the Author
This is an extremely well written paper that describes the results of an engaging experiment, which characterizes across a number of dimension the properties of gamma oscillations in the hippocampus of birds (chicks). In all sincerity, there is very little to criticize about this paper. I only have a few minor suggestions for improvement.

(But I do have one caveat. I have little to no expertise in the quantitative analysis of EEG (local field potential) recordings or in vitro slice preparations. I am not comfortable evaluating these aspects of the paper.)

1. On line 70, I am not sure “convergent functions” is the right phrase to use here. One cannot exclude that similar function is derived from a common ancestor and the divergent cytoarchitecture came

later.
2. Some readers will be curious to know the logic behind using two different breeds of chickens.
3. There is some grammatical hiccup on line 141 ("For recordings slice…").
4. For the pharmacological manipulations, some readers might want to know a little more about why the particular concentrations were used.

That is really all I have; a very nice paper.

Reviewer: 2 (Martin Stacho, Ruhr-University Bochum, Germany)

Comments to the Author
Deheerendra et al. performed an in-vitro study of the avian hippocampus with the focus on demonstration of gamma oscillations in this brain structure in birds. The authors indeed found that gamma oscillations can be induced in the chick hippocampal slices by carbachol - an acetylcholine agonist; and are dependent on GABA-A, AMPA and M1 receptor conductivities. They pointed out that these characteristics are similar to gamma oscillations found in the mammalian hippocampus and therefore concluded that the avian and mammalian hippocampi utilize similar microcircuits which are the basis of the comparable function of the hippocampus in these two species. This is an excellent and well-performed study and the results and the manuscript are easy to follow. I very much appreciate this work since it is an extremely valuable contribution to our understanding of the network properties of the avian hippocampus. This is certainly one of the most important studies in this field as it is a milestone in the comparative hippocampal neurobiology which provides a good fundament for future research of the hippocampal circuitry. I have following minor comments, which I hope will help the authors and will further improve the manuscript:

First of all, I am struggling a little bit with the conclusion about the underlying microcircuits. In this study, the authors performed extracellular recordings and investigated the changes in LFP during pharmacological manipulations of the network. These experiments provide us with information about the receptors and neurotransmitters involved in the generation of the gamma oscillations. Given the neurotransmitters, one can also to some degree infer the types of neurons involved (e.g. glutamatergic, gabaergic, cholinergic..). However, at least to me, these data are not sufficient to infer the underlying circuitry or even the microcircuitry. Several times in the manuscript, the authors do talk about the microcircuitry and its similarity to the mammalian hippocampus, unfortunately without providing any details about this idea. For me, this conclusion of the manuscript is not a real conclusion but rather a brave speculation. Although the authors point out the Golgi studies of Tömböl et al. in which connections between GABAergic and pyramidal neurons have been demonstrated, an idea of a proper hippocampal circuitry in birds is still not established. I would ask the authors to present their ideas about the underlying circuitry more clearly and little bit more detailed. Nevertheless, the conclusion that the avian and mammalian hippocampi might perform comparable operations (based on the presence of gamma oscillations) is very interesting and important outcome of the manuscript.

Furthermore, it is written in the manuscript that the microcircuitry underlying the hippocampal gamma oscillations is well known in the mammalian hippocampus. Also here, no details are provided. It would be great if authors could elaborate more on this point as it would make the statements about the circuits much more clear and would be also necessary for the comparative speculations. With this regard, I would also kindly ask the authors to include more hippocampus-related citations into their manuscript. Especially the statement that parvalbumin-positive GABAergic neurons are important for gamma oscillations in CA3 region of the mammalian hippocampus is supported by citations of Cardin et al. (2009) and Sohal et al. (2009). However, to my knowledge, these cited studies were performed in the rodent neocortex but not hippocampus. Please include also citations dealing with hippocampus as the sentence is otherwise confusing and the citation de facto incorrect. They are many different hippocampal studies which could be cited here (incl. e.g. Fuchs et al. 2007, Mann et al. 2005; Gloveli et al. 2005 etc.). In addition, do the authors have any evidence for the presence of fast-spiking /parvalbumin-positive interneurons within the avian hippocampus - the key players of gamma oscillations in mammals?

The regional differences in the gamma oscillations are very interesting. Especially the similarities between the CA3 and the ventromedial region of the avian hippocampus pointed out by the authors. I think, it might be also important to mention that CA3 is not the only generator of the gamma oscillations in the mammalian hippocampus. It seems that other regions such as CA1, medial EC or subiculum can independently generate gamma band oscillations. This then does not exclude the presence of gamma oscillations in other subregions from the discussion and the comparison and it of course still fits to the data of the present study. Without proposing any further experiments to be performed for this manuscript, I was just wondering whether the authors tried to record simultaneously from two different hippocampal subregions? Such recordings could provide information about the communication between the subregions and/or whether the avian hippocampus contains independent gamma generators.

As mentioned by the authors, gamma oscillations are present not only in the hippocampus but also in the neocortex and in birds they were extensively investigated in the midbrain. Given that gamma oscillations are

therefore a quite ubiquitous phenomenon, it is important to elaborate the question, to what extent the authors found a genuine hippocampal feature or rather a general principle of (fore)brain networks. In both cases the implications of the present findings are extremely fascinating. It would be nice if the authors could somehow more disentangle (or comment on) these two options (if there is anything either in the literature or in the authors data).

In case the authors have the unfiltered data, in would be worth to see if there are also theta oscillations (another feature of the hippocampus) present in their LFPs and how they are related to the gamma band oscillations. Theta and gamma oscillations are simultaneously present in the mammalian hippocampus during high levels of acetylcholine, and their cross-frequency coupling is an important and maybe distinctive feature.

With regard to the puzzling increase in the gamma power after D-AP5 administration in this study, it might be interesting to note that blocking of NMDA-receptors in neocortex and hippocampus in-vivo using ketamine or MK-801 also increases the power of gamma oscillations (Pinault, 2008; Lazarewicz et al., 2010).

Last small thing: I do not get how the conclusion in line 293 that the mAchRs are selectively expressed at the excitatory neurons can be made from the previous sentence.

Authors' Response 28 October 2017

Dear Drs. Foxe and Bolam,

Thank you very much for the constructive criticism on our manuscript. We have gone through your and the reviewers' comments, and have made changes to the manuscript to improve clarity and answer questions. The major changes in the manuscript have been marked in bold, as instructed. Our Responses to the different comments are inserted in blue below:

Your manuscript was reviewed by two external reviewers as well as by the editors. The reviews collectively indicate that your experiments generated new and important information and all concerned found the paper to be a very compelling piece of work, well written and clearly articulated. However, there are several (mostly minor) issues that need to be resolved before we can proceed further with your manuscript for publication in EJN. The reviewers have provided clear points to which you should respond in your revision. In addition, please also attend to the following issues which have been identified by the editorial office:

1) Be sure to provide the full name of the ethics committee that approved the work

The full name was there in the original, but we have made it less ambiguous.

2) Please specify the % isoflurane used to anesthetize chicks prior to decapitation

We have added this information to the methods.

3) Please be very explicit about the number of animals used in the experiment

Total numbers of birds have been added to the methods.

4) A scale bar is needed for fig 3a

We have added a scale bar and changed the image (see response to reviewer 2).

5) Replace your barcharts with scatterplots or some other more informative hybrid depictions - see http://onlinelibrary.wiley.com/doi/10.1111/ejn.13400/epdf

We have added individual data to the different graphs, to indicate the real variation in the data.

Reviews:

Reviewer: 1

Comments to the Author
This is an extremely well written paper that describes the results of an engaging experiment, which characterizes across a number of dimension the properties of gamma oscillations in the hippocampus of birds (chicks). In all sincerity, there is very little to criticize about this paper. I only have a few minor suggestions for improvement.

(But I do have one caveat. I have little to no expertise in the quantitative analysis of EEG (local field potential) recordings or in vitro slice preparations. I am not comfortable evaluating these aspects of the paper.)

1. On line 70, I am not sure “convergent functions” is the right phrase to use here. One cannot exclude that similar function is derived from a common ancestor and the divergent cytoarchitecture came later.

The reviewer is of course correct. We have changed this to a more neutral “similar” function.

2. Some readers will be curious to know the logic behind using two different breeds of chickens.

The reason was purely practical: When we first started this work, we found a breeder of Wellsummer chickens relatively nearby, and started using those. However, this is a seasonally breeding chicken breed, and availability of chicks was unreliable, so for the second part of the study (a year later), we switched to a commercial supplier, with more reliable supply, but more complicated logistics. We have added a few words to the methods to clarify this.

3. There is some grammatical hiccup on line 141 (“For recordings slice…”).

Thank you, the grammar has been fixed.

4. For the pharmacological manipulations, some readers might want to know a little more about why the particular concentrations were used.
We used the concentrations that are routinely used in the mouse experiments carried out in our lab.

That is really all I have; a very nice paper.

Thank you!

Reviewer: 2

Comments to the Author
Deheerendra et al. performed an in-vitro study of the avian hippocampus with the focus on demonstration of gamma oscillations in this brain structure in birds. The authors indeed found that gamma oscillations can be induced in the chick hippocampal slices by carbachol - an acetylcholine agonist; and are dependent on GABA-A, AMPA and M1 receptor conductivities. They pointed out that these characteristics are similar to gamma oscillations found in the mammalian hippocampus and therefore concluded that the avian and mammalian hippocampi utilize similar microcircuits which are the basis of the comparable function of the hippocampus in these two species. This is an excellent and well-performed study and the results and the manuscript are easy to follow. I very much appreciate this work since it is an extremely valuable contribution to our understanding of the network properties of the avian hippocampus. This is certainly one of the most important studies in this field as it is a milestone in the comparative hippocampal neurobiology which provides a good fundament for future research of the hippocampal circuitry. I have following minor comments, which I hope will help the authors and will further improve the manuscript:

Thank you for the kind words.

First of all, I am struggling a little bit with the conclusion about the underlying microcircuits. In this study, the authors performed extracellular recordings and investigated the changes in LFP during pharmacological manipulations of the network. These experiments provide us with information about the receptors and neurotransmitters involved in the generation of the gamma oscillations. Given the neurotransmitters, one

can also to some degree infer the types of neurons involved (e.g. glutamatergic, gabaergic, cholinergic..). However, at least to me, these data are not sufficient to infer the underlying circuitry or even the microcircuitry. Several times in the manuscript, the authors do talk about the microcircuitry and its similarity to the mammalian hippocampus, unfortunately without providing any details about this idea. For me, this conclusion of the manuscript is not a real conclusion but rather a brave speculation. Although the authors point out the Golgi studies of Tömböl et al. in which connections between GABAergic and pyramidal neurons have been demonstrated, an idea of a proper hippocampal circuitry in birds is still not established. I would ask the authors to present their ideas about the underlying circuitry more clearly and little bit more detailed. Nevertheless, the conclusion that the avian and mammalian hippocampi might perform comparable operations (based on the presence of gamma oscillations) is very interesting and important outcome of the manuscript.

The original conclusion makes clear that we never meant to imply that we have completely worked out the circuitry, just because we have a similar pharmacology in birds and mammals. We have changed the wording thoughout the manuscript to make clear that the similarity in pharmacology leads to the hypothesis that the underlying circuits are also similar, but that it requires further work on single cells (intracellular and/or patch-clamp) to fully work out the circuits involved.

Furthermore, it is written in the manuscript that the microcircuitry underlying the hippocampal gamma oscillations is well known in the mammalian hippocampus. Also here, no details are provided. It would be great if authors could elaborate more on this point as it would make the statements about the circuits much more clear and would be also necessary for the comparative speculations.

We have added details of the hippocampal micro-circuitry to the introduction.

With this regard, I would also kindly ask the authors to include more hippocampus-related citations into their manuscript. Especially the statement that parvalbumin-positive GABAergic neurons are important for gamma oscillations in CA3 region of the mammalian hippocampus is supported by citations of Cardin et al. (2009) and Sohal et al. (2009). However, to my knowledge, these cited studies were performed in the rodent neocortex but not hippocampus. Please include also citations dealing with hippocampus as the sentence is otherwise confusing and the citation de facto incorrect. They are many different hippocampal studies which could be cited here (incl. e.g. Fuchs et al. 2007, Mann et al. 2005; Gloveli et al. 2005 etc.).

We have added more hippocampal-relevant citations to the text.

In addition, do the authors have any evidence for the presence of fast-spiking /parvalbumin-positive interneurons within the avian hippocampus - the key players of gamma oscillations in mammals?

We have indeed stained chick hippocampal slices using an antibody against parvalbumin, and have found many parvalbumin-positive cells in the hippocampus. We do not yet know whether these have the same physiological properties as mammalian parvalbumin-positive cells. We have added a photomicrograph of this staining to Figure 3 as an illustration and evidence of presence of this kind of interneurons.

The regional differences in the gamma oscillations are very interesting. Especially the similarities between the CA3 and the ventromedial region of the avian hippocampus pointed out by the authors. I think, it might be also important to mention that CA3 is not the only generator of the gamma oscillations in the mammalian hippocampus. It seems that other regions such as CA1, medial EC or subiculum can independently generate gamma band oscillations.

We have pointed out in our discussion that other areas in the mammalian hippocampal formation generate gamma rhythms, and that further work is necessary in the bird hippocampal formation to work out which regions generate gamma and which follow.

This then does not exclude the presence of gamma oscillations in other subregions from the discussion and the comparison and it of course still fits to the data of the present study. Without proposing any further experiments to be performed for this manuscript, I was just wondering whether the authors tried to record simultaneously from two different hippocampal subregions? Such recordings could provide information about the communication between the subregions and/or whether the avian hippocampus contains independent gamma generators.

EJN European Journal of Neuroscience FENS Federation of European Neuroscience Societies

Unfortunately, we have not yet tried simultaneous recordings from different sub-regions of the hippocampus. This is definitely in the plans for future projects.

As mentioned by the authors, gamma oscillations are present not only in the hippocampus but also in the neocortex and in birds they were extensively investigated in the midbrain. Given that gamma oscillations are therefore a quite ubiquitous phenomenon, it is important to elaborate the question, to what extent the authors found a genuine hippocampal feature or rather a general principle of (fore)brain networks. In both cases the implications of the present findings are extremely fascinating. It would be nice if the authors could somehow more disentangle (or comment on) these two options (if there is anything either in the literature or in the authors data).

Of course, we have no information on gamma oscillation generators elsewhere in the avian forebrain, but we have added a paragraph speculating that it must exist elsewhere, as well as speculating about the possible implications of the spatial distribution of gamma power within the avian hippocampal formation.

In case the authors have the unfiltered data, in would be worth to see if there are also theta oscillations (another feature of the hippocampus) present in their LFPs and how they are related to the gamma band oscillations. Theta and gamma oscillations are simultaneously present in the mammalian hippocampus during high levels of acetylcholine, and their cross-frequency coupling is an important and maybe distinctive feature.

Unfortunately, the filter settings on our rigs excluded oscillations below 10Hz, so that we would have missed any theta oscillations, if they had been present. This will definitely be changed for future studies.

With regard to the puzzling increase in the gamma power after D-AP5 administration in this study, it might be interesting to note that blocking of NMDA-receptors in neocortex and hippocampus in-vivo using ketamine or MK-801 also increases the power of gamma oscillations (Pinault, 2008; Lazarewicz et al., 2010).

We have made a note of this in the discussion and added the references.

Last small thing: I do not get how the conclusion in line 293 that the mAchRs are selectively expressed at the excitatory neurons can be made from the previous sentence.

We have clarified our reasoning. The excitatory neurons are just one example of a population of neurons that might express them, based on the reference that follows it. We have rewritten the sentence to clarify this.
